# Trofinetide—a new chapter in rett syndrome’s treatment

**DOI:** 10.3389/fphar.2023.1284035

**Published:** 2023-11-16

**Authors:** Muhammad Furqan

**Affiliations:** Department of Medicine, Mayo Hospital, King Edward Medical University, Lahore, Punjab, Pakistan

**Keywords:** rett syndrome (RTT), x linked, neurodegenrative disease, intellectual disabililties, MECP 2 gene

## Abstract

Trofinetide is the first drug approved by the FDA to treat Rett Syndrome in children aged 2 years or above. The drug significantly improved Rett syndrome behavioral scores Rett syndrome behavioral questionnaire in clinical studies. Although further research is needed to assess potential adverse events, Trofinetide’s notable efficacy signifies a significant advancement in Rett syndrome treatment, offering a new therapeutic avenue with the potential to ameliorate the condition.

## Introduction

Rett syndrome is a rare (one in 10,000 live births) sporadic neurodevelopmental disorder characterized by normal early growth and development followed by regression in previously acquired activities, primarily affecting motor, cognitive, and communication skills (Valenti and Vacca, 2023). It is one of the most prevalent genetic causes of intellectual disability in females, second to Down syndrome (Borloz et al., 2021). All cases of Rett syndrome are almost attributed to the *de novo* mutation in the methyl CpG binding protein 2 or MECP2 on the X chromosome. The mechanism by which MECP2 causes intellectual disability is not yet fully understood. However, two of the potential hypotheses are that the lack of MECP2 results in the defective synaptic maturation of the cortex or that it hampers the metabolism of brain cholesterol, leading to irregular neuronal growth (Chahil and Bollu, 2023). There is yet no known way of preventing this disease. However, the United States Food and Drug Administration (FDA) approved Trofinetide, in March 2023 for treating Rett Syndrome in children aged 2 years or older (FDA, 2023). This is a landmark step in treating Rett Syndrome as it is the first and only approved treatment. The LAVENDER trial (Study of Trofinetide for the Treatment of Girls and Women with Rett Syndrome) is the basis for this approval. This 12-week randomized, double-blind, placebo-controlled, parallel-group study investigated the safety, effectiveness, and tolerability of Trofinetide in addressing Rett syndrome among 187 female participants aged 5–20 years (ACADIA Pharmaceuticals Inc, 2022). The co-primary outcomes of this study were the change in RSBQ total score from baseline to week 12 and the CGI-I score at week 12 (Neul et al., 2022).

Trofinetide is a water-soluble analogue of glycine-proline-glutamate (GPE). GPE is an N-terminal tripeptide product of the cleavage of insulin-like growth factor 1 (IGF-1) found in the brain (Hudu et al., 2023) and is neuroprotective at minimal doses. The mechanism of action of Trofinetide for treating Rett syndrome is not entirely understood, but it is thought to work on the same principle as GPE but with a longer half-life (Bickerdike et al., 2009). Many studies have indicated that Trofinetide exerts its effect by enhancing synaptic activities, restoring synaptic structure, suppressing the effects of inflammatory compounds in the brain, increasing antioxidative reactions, decreasing injury-triggered cell death, and increasing the presence of IGF-1 in the central nervous system (Hudu et al., 2023). According to the results of the LAVENDER trial, this drug has demonstrated a promising improvement in the Rett syndrome behavioral questionnaire (RSBQ) total score compared to the placebo. The average change in the RSBQ total score from baseline to week 12 was recorded as −5.1 (with a standard deviation of 0.99) for the Trofinetide group and −1.7 (with a standard deviation of 0.98) for the placebo group. According to the analysis using the mixed-effect model for repeated measures (MMRM), there was a statistically significant difference in the change from baseline to week 12 in the RSBQ total score between Trofinetide and placebo. Trofinetide showed a significantly greater decrease (−4.9 (0.94)) compared to placebo (−1.7 (0.90)). The treatment difference between Trofinetide and placebo was −3.1 (1.30) (95% confidence interval (CI), −5.7 to −0.6; *p* = of 0.0175; Cohen’s d effect size, 0.37). The mean (s.e.m.) CGI-I scores at week 12 in the Trofinetide and placebo groups were 3.5 (0.08) and 3.8 (0.06), respectively. The MMRM analysis revealed a statistically significant improvement with Trofinetide compared to placebo at week 12, with a treatment difference of −0.3 (0.10) (95% CI, −0.5 to −0.1; *p* = 0.0030; Cohen’s d effect size, 0.47). Regarding the coprimary endpoints, subgroup analyses exhibited a consistent advantage of Trofinetide over placebo, regardless of age, initial RSBQ severity, and classification of MECP 2 mutation severity (Neul et al., 2023). These significant improvements in the RSBQ total score and coprimary endpoints contributed to the FDA’s accelerated approval of this drug.

However, it is not devoid of side effects and requires attentive surveillance. Among the observed treatment-related adverse events (TEAEs) experienced by patients, diarrhea, vomiting, fever, anxiety, seizures, fatigue, nasopharyngitis, and reduced appetite were noted, with diarrhea and vomiting being the most prevalent. Eighteen participants discontinued treatment due to a TEAE (Trofinetide: 16 participants (17.2%); placebo: 2 participants (2.1%)), with diarrhea being the primary reason for discontinuation (Trofinetide: 12 participants (12.9%)). In long-term studies, approximately 2.2% of patients discontinued Trofinetide treatment due to weight loss (Acadia Pharmaceuticals, 2023). These unfavorable health effects constitute the primary basis for the essential boxed warning that alerts healthcare professionals about the potential for diarrhea and associated weight loss.

Although this drug has yet to undergo testing in more extensive trials for potential adverse events, the increase in RSBQ total score shows its significant advantage in treating Rett Syndrome. The majority of these adverse outcomes demonstrate mild to moderate severity. While this medication does not entirely eradicate Rett syndrome and cannot be classified as a definitive cure, its impressive effectiveness and innovative impact suggest the potential for introducing a new treatment paradigm for Rett syndrome.

## Data Availability

The original contributions presented in the study are included in the article/Supplementary material, further inquiries can be directed to the corresponding author.
